# Supplementary material for: Infants are superior in implicit crossmodal learning and use other learning mechanisms than adults
Source: eLife. 2017 Sep 26;6:e28166. doi: 10.7554/eLife.28166 (PMC5662286; doi:10.7554/eLife.28166)
Supplement: Transparent reporting form [file elife-28166-transrepform.pdf]

eLIFE
1st Floor
24 Hills Road
Cambridge CB2 1JP, UK
P 01223 855340
W elifesciences.org
T @elife

# *eLife's* transparent reporting form

We encourage authors to provide detailed information *within their submission* to facilitate the interpretation and replication of experiments. If you have any questions, please contact us: editorial@elifesciences.org.

## Sample-size estimation

- You should state whether an appropriate sample size was computed when the study was being designed
- You should state the statistical method of sample size computation and any required assumptions
- If no explicit power analysis was used, you should describe how you decided what sample (replicate) size (number) to use

Please outline where this information can be found within the submission (e.g., page numbers or figure legends), or explain why this information doesn't apply to your submission:

Sample size was selected based on previous studies investigating typical sensory mismatch ERP effects (Experiment 1a, 2a and 2b). For Experiment 1a we expected a typical dropout rate of about 50 % (see manuscript, line 132) for infant EEG data sets. Sample size was doubled for 1b compared to 2a/b due to the shorter recording time (same as for infants in experiment 1a).

In each experiment we found a significant sensory deviant effects, and in Experiment 1a and 2b we found ERP deviant effects for rare combined crossmodal stimuli indicating that our experiments had a sufficient power to reveal ERP effects of interest.

Note, that the ERP sensory deviant effect was replicated in four experiments and the ERP deviant effect for newly combined stimuli in two experiments (according the hypothesis put forward based on the literature).

## Replicates

- You should report how often each experiment was performed
- You should include a definition of biological versus technical replication
- The data obtained should be provided and sufficient information should be provided to indicate the number of independent biological and/or technical replicates
- If you encountered any outliers, you should describe how these were handled
- Criteria for exclusion/inclusion of data should be clearly stated

eLife Sciences Publications, Ltd is a limited liability non-profit non-stock corporation incorporated in the State of Delaware, USA, with company number 5030732, and is registered in the UK with company number FC030576 and branch number BR015634 at the address 1st Floor, 24 Hills Road, Cambridge CB2 1JP | August 2014

eLIFE
1st Floor
24 Hills Road
Cambridge CB2 1JP, UK
P 01223 855340
W elifesciences.org
T @elife

- High-throughput sequence data should be uploaded before submission, with a private link for reviewers provided (these are available from both GEO and ArrayExpress)

Please outline where this information can be found within the submission (e.g., page numbers or figure legends), or explain why this information doesn't apply to your submission:

Each of the four experiment was performed once. However, it has to be noted that we replicated the sensory deviant ERP response in all four experiments in overlapping ERP time epochs (see "Results" section and further discussed in the "Discussion" section). Moreover we replicated the ERP deviant effect for rare combinations in two independent groups (Experiment 1a and 2b) again at similar time epochs.

Criteria for exclusion of data for Experiment 1 and 1b: "Methods", line 606
Criteria for exclusion of data for Experiment 2a and 2b: "Methods", line 661

**Statistical reporting**

- Statistical analysis methods should be described and justified
- Raw data should be presented in figures whenever informative to do so (typically when N per group is less than 10)
- For each experiment, you should identify the statistical tests used, exact values of N, definitions of center, methods of multiple test correction, and dispersion and precision measures (e.g., mean, median, SD, SEM, confidence intervals; and, for the major substantive results, a measure of effect size (e.g., Pearson's r, Cohen's d)
- Report exact p-values wherever possible alongside the summary statistics and 95% confidence intervals. These should be reported for all key questions and not only when the p-value is less than 0.05.

Please outline where this information can be found within the submission (e.g., page numbers or figure legends), or explain why this information doesn't apply to your submission:

N of Experiment 1a: "Methods", line 534
N of Experiment 1b: "Methods", line 571
N of Experiment 2a/2b: "Methods", line 661

Statistical Analysis of Experiment 1a: "Methods", page 24/25
Statistical Analysis of Experiment 1b and 2a/2b: Methods", page 28/29

Statistical values of Experiment 1a: "Results", page 7 -9
Statistical values of Experiment 1b: "Results", page 9/10
Statistical values of Experiment 2a: "Results", page 11/12
Statistical values of Experiment 2b: "Results", page 12-14

eLife Sciences Publications, Ltd is a limited liability non-profit non-stock corporation incorporated in the State of Delaware, USA, with company number 5030732, and is registered in the UK with company number FC030576 and branch number BR015634 at the address 1st Floor, 24 Hills Road, Cambridge CB2 1JP | August 2014

(For large datasets, or papers with a very large number of statistical tests, you may upload a single table file with tests, Ns, etc., with reference to page numbers in the manuscript.)

**Additional data files ("source data")**

- We encourage you to upload relevant additional data files, such as numerical data that are represented as a graph in a figure, or as a summary table
- Where provided, these should be in the most useful format, and they can be uploaded as "Source data" files linked to a main figure or table
- Include model definition files including the full list of parameters used
- Include code used for data analysis (e.g., R, MatLab)
- Avoid stating that data files are "available upon request"

Please indicate the figures or tables for which source data files have been provided:

Numerical data are reported in the tables with a precision measure.

Figures show ERP time – voltage plots and topographical representations ("Results").
